# Understanding the factors that affect the appropriateness of rheumatology referrals

**DOI:** 10.1186/s12913-021-07036-5

**Published:** 2021-10-19

**Authors:** Eline van den Broek-Altenburg, Adam Atherly, Nick Cheney, Teresa Fama

**Affiliations:** 1 Department of Radiology, Larner College of Medicine, University of Vermont, 89 Beaumont Ave, Burlington, VT 05405 USA; 2 Center for Health Services Research, Larner College of Medicine, University of Vermont, Burlington, USA; 3 Department of Computer Science, University of Vermont, Burlington, USA; 4 Department of Rheumatology, Central Vermont Medical Center, Berlin, USA

**Keywords:** Value-based healthcare, Access to care, Referrals, Electronic health records data, Intermeasure reliability

## Abstract

**Background:**

Reducing inappropriate referrals to specialists is a challenge for the healthcare system as it seeks to transition from volume to value-based healthcare. Given the projection of a severe shortage of rheumatologists in the near future, innovative strategies to decrease demand for rheumatology services may prove more fruitful than increasing the supply of rheumatologists. Efforts to increase appropriate utilization through reductions in capacity may have the unintended consequence of reducing appropriate care as well. This highlights the challenges in increasing the appropriate use of high cost services as the health system transitions to value based care. The objective of this study was to analyze factors affecting appropriateness of rheumatology services.

**Methods:**

This was a cross-sectional study of patients receiving Rheumatology services between November 2013 and October 2019. We used a proxy for “appropriateness”: whether or not there was any follow-up care after the first appointment. Results from regression analysis and physicians’ chart reviews were compared using an inter-rater reliability measure (kappa). Data was drawn from the EHR 2013–2019.

**Results:**

We found that inappropriate referrals increased 14.3% when a new rheumatologist was hired, which increased to 14.8% after wash-out period of 6 months; 15.7% after 12 months; 15.5% after 18 months and 16.7% after 18 months. Other factors influencing appropriateness of referrals included severity of disease, gender and insurance type, but not specialty of referring provider.

**Conclusions:**

Given the projection of a severe shortage of rheumatologists in the near future, innovative strategies to decrease demand for rheumatology services may prove more fruitful than increasing the supply of rheumatologists. Innovative strategies to decrease demand for rheumatology services may prove more fruitful than increasing the supply of rheumatologists. These findings may apply to other specialties as well. This study is relevant for health care systems that are implementing value-based payment models aimed at reducing inappropriate care.

## Background

Using specialist-trained physicians appropriately is a challenge for the healthcare system. Specialists have greater knowledge of particular diseases and conditions and can, for some patients, provide superior care to primary care physicians [1]. But specialists are also more expensive [2, 3], use more ancillary services [4], and often provide care outside their narrow provider specialty [5]. And, beyond those issues, the availability of many specialists is limited, with projections of shortages for many specialist types [6, 7].

The appropriate use of specialists is not a primary problem for many healthcare systems in a volume-based system. Under Fee-For-Service (FFS), reimbursement rates were higher for specialists than primary care providers were, so “over” utilization of specialists generated higher revenues for the system. But as the healthcare system begins to transition away from volume and toward value based care [8–12], the financial incentives for the use of specialty care are also transitioning [13–15], leading to a need for more effective targeting of patients to specialists.

Yet most specialists actually have limited control over the demand for their services. Patients do sometime self-refer to specialists [16–18], but many specialists receive referrals from primary care providers [19]. To effectively target patients and resolve potential or actual shortages of services, it is therefore necessary to understand what patient, provider and clinic factors effect patient referrals.

One additional complication is the possibility of “supplier induced demand” [20–22]. This is the idea that the number of referrals may be endogenous to the number of providers. Given that there is discretion in when a referral is “needed”, the availability of capacity may induce inappropriate utilization.

One specialty where the referral challenge is particularly acute is rheumatology. An aging population and declining work force are creating shortages of providers in some areas, with demand for rheumatology services already exceeding supply by about 13%, or approximately 700 full-time equivalent (FTE) rheumatologists [1]. By 2030, demand is expected to outpace supply by 4133 FTEs (102%).

The ability to increase the supply of rheumatologists is limited by a number of factors, including restrictions on fellowship program positions, inadequate fellowship program fill-rates, a trend toward part-time work, and challenges surrounding international medical graduates’ ability to remain in the US [23]. Given the projection of a severe shortage of rheumatologists in the near future, innovative strategies to decrease the demand for rheumatology services by targeting services to patients who will benefit the most may prove more fruitful than increasing the supply of rheumatologists.

A review of the literature on interventions that address specialty referral management found that about one-third of referrals to rheumatologists are unnecessary or inappropriate [24]. Several studies have evaluated interventions to improve the quality of referrals to rheumatologists. The most effective interventions combine iterative rheumatologist feedback to the referring provider, along with clear referral criteria or evidence-based guidelines [24–26]. A study by Lohr et al. compared the quality of referrals from physicians, physician assistants and nurse practitioners and found that referrals from physicians were of better quality (measured by a number of factors) and were less likely to be unnecessary [27]. Little is known regarding other (physician) factors that affect the appropriateness or quality of referrals.

The objective of this study was to identify factors that affect the appropriateness of rheumatology referrals from primary care providers. We used a quantitative analysis using quasi-experimental design and regression analysis. To understand whether induced demand for rheumatology care leads to an increase in inappropriate referrals, we looked at the effect of the start of an additional rheumatologist on the appropriateness of referrals. We looked at this effect 2 months after the start of the rheumatologist (assuming that there would be at least 2 months between a referral and a first appointment) and during a washout period of 6 months, 12, 18 and 24 months, enabling us to analyze the referrals pattern over a period of time.

## Methods

All methods were carried out in accordance with relevant guidelines and regulations. The institutional review boards of the Central Vermont Medical Center and the University of Vermont approved this study. We used de-identified data from electronic health records; no consent to participate was needed or received for this study.

### Data

Our primary source of data for this study was Electronic Health Records (EHRs). Our sample included all patients who visited the Central Vermont Medical Center (CVMC) between November 1, 2013 and October 31, 2019 where the EHR was used to record all data. Our sample inclusion criteria was an appointment in CVMC Rheumatology, providing an initial sample size of 3387 referrals. Exclusion criteria included: patients who did not complete their initial Rheumatology appointment and those with no-shows and cancellations. We also limited the final data set to be one occurrence per unique patient during the study period (*n* = 2765). Approximately a third of referrals were internal providers (*n* = 916) and two thirds were external (*n* = 1849). There were three rheumatology specialists handling all patients in our study and none of the referrals were denied. The three rheumatologists were all experienced, at least 10 years post-fellowship. They were all full-time clinicians, two female and one male and all employed by the hospital.

### Design and study setting: defining appropriate referral

To determine whether a referral was appropriate, three Rheumatology rheumatologists at the medical center performed chart reviews (*n* = 1020 and made independent assessments about appropriateness. We had a time period for collecting the data for the chart review and we reviewed every new referral request over a select time period. That allowed us to manually track the outcomes of the referral much more accurately than using the EHR. We had to make a judgment of appropriate or not appropriate prior to the referral being scheduled. We would not have been able to do this if we just relied on the EHR data – the referral would have already been placed.

The inter-rater reliability of the comparison was calculated using Cohen’s kappa (K). Kappa measures the concordance between two different measures of two raters. It adjusts for the rate of agreement expected by random chance using equation (1):

1 $$ \mathrm{K}=\frac{\mathrm{P}0-\mathrm{P}\mathrm{e}}{1-\mathrm{P}\mathrm{e}}=1-\frac{1-\mathrm{P}0}{1-\mathrm{P}\mathrm{e}} $$

where P_0_ is the observed agreement among raters, and P_e_ is the hypothetical probability of chance agreement, which is defined by using the observed data to calculate the probabilities of each observer randomly seeing each referral. If the raters are in complete agreement, then K = 1.

The results of the chart reviews, indicating which referrals were “appropriate” according to clinician’s judgment, were also compared to the proxy measure from the EHR data using an inter-measure reliability measure.

We also used proxy for “appropriateness” of referrals: whether or not there was any follow-up care for the patient after the first rheumatologist visit. The logic of this measure is that if the patient required continuing care from the rheumatologist, the referral was appropriate. If no continuing care was offered, then the referral was not appropriate.

Analytically, we used logit models to identify factors affecting the appropriateness of referral. Control variables included patient characteristics (age, gender, CCI and county), insurance status and provider characteristics (whether the referring person was a physician or Advanced Practice Provider (APP) and service line (family practice, internal medicine, other specialty or naturopath). In addition, we used 10 years of discharge diagnoses for patients with Rheumatology appointments to develop a Charlson Comorbidity Index (CCI) based on their ICD-10 codes reported in the EHR during this 10-year period. The dependent variable in the analysis was whether or not the referral was identified as appropriate.

We used a Difference-in-Differences (DiD) model to analyze if there were changes in referral-appropriateness when the new rheumatologist started, hypothesizing that increased supply may lead to induced demand.

The difference-in-differences model is estimated as follows in equation (2):

2 $$ p\left( Inappropriate\ referral\right)= Constant+ IR\ {\beta}_1+ rheumatologist\ {\beta}_2+ IR\ x\  rheumatologist\ {\beta}_3+ Control\ Variables\ {\beta}_{Control}+u $$

where IR = 1 if the patient was internally referred, according to the EHR, and 0 if a patient was identified as externally referred, and rheumatologist = 1 after the date the extra rheumatologist started and 0 otherwise. The coefficient of the treatment variable, β1, is the estimated mean difference in inappropriate referrals between the internally and externally referred patients. It represents whatever “baseline” differences existed between the groups before the new rheumatologist was added. β2 is the expected mean change in outcome from before to after the start of the new rheumatologist. The rheumatologist effect on inappropriate referrals is measured by β3, the coefficient on the interaction term (IR x rheumatologist) which measures the “difference-in-differences”. It tells us whether the expected mean change in the probability of having an inappropriate referral from before to after the rheumatologist started was different in the two groups. We used marginal effects for all models, which measure the discrete change.

## Results

Descriptive statistics for the sample are provided in Table 1. Of the additional scheduled appointments in the EHR during the research period, 70% were completed, 9% were cancelled by the patient, 13% were rescheduled, 4% of patients did not show up for the appointment and 4% were office cancellations. After the non-completed referrals were excluded, the total sample size was 2765 patients, of which about a third internally referred (*n* = 916) and two thirds were externally referred (*n* = 1849).

**Table 1:** Summary Statistics by Appropriateness of Referral

	Appropriate (***n*** = 1921) % (n)	Inappropriate (***n*** = 844) % (n)
**Age (yrs)** ^a^		
*< 40*	13.7 (264)	19.1 (161)
*40–49*	13.8 (265)	15.2 (128)
*50–59*	23.3 (448)	21.8 (184)
*60–69*	25.0 (481)	23.2 (196)
*70–79*	16.3 (313)	12.9 (109)
*80+*	7.8 (150)	7.8 (66)
**Gender** ^a^		
*Male*	30.6 (588)	26.5 (224)
*Female*	69.4 (1333)	73.5 (620)
**Insurance Type**^a^		
*Medicare*	10.2 (196)	14.1 (119)
*Medicaid*	45.2 (869)	41.1 (347)
*Private or other*	44.6 (856)	44.8 (378)
**CCI**^a^		
*Zero*	39.3 (369)	55.9 (254)
*One*	31.7 (297)	24.4 (111)
*Two*	13.1 (123)	6.8 (31)
*Three or More*	15.9 (149)	12.8 (58)
***Provider Characteristics***		
**Referred by rheumatologist or DO**		
*Family Practice*	0.66 (654)	0.66 (257)
*Internal Medicine*	0.26 (252)	0.23 (89)
*Specialty*	0.08 (77)	0.11 (44)
*Naturopath*	0 (0)	0 (0)
**Referred by APP or other**		
*Family Practice*	0.33 (314)	0.33 (148)
*Internal Medicine*	0.03 (29)	0.03 (13)
*Specialty*	0.04 (33)	0.04 (16)
*Naturopath*	0.02 (16)	0.03 (12)
*Not Specified other*	0.58 (546)	0.58 (256)

^a^Chi-square, *p* < .05

After comparing rheumatologist’s chart reviews (*n* = 102) for the appropriateness of referrals, we found that there was 84.3% actual agreement and 72.2% expected agreement. The inter-rater reliability measure kappa, therefore, was 0.68, which represents “substantial agreement” [10]. We found that there was 88% agreement between the reviewers and the follow-up proxy measure in the EHR data. The inter-measure kappa (comparing the reviewer rating and whether a follow-up was scheduled) is 0.65, which again represents substantial agreement.

There were more females with inappropriate referrals (73.5%) than with appropriate referrals (69.4%). A higher comorbidity index was associated with a greater likelihood of an appropriate referral (i.e., for CCI = 1, appropriate referrals 31.7% versus 24.4% for inappropriate referrals; 13.1% versus 6.8% for CCI = 2 and 15.9% versus 12.8% for CCI > 2). There were fewer Medicare enrollees (10.2% versus 14.1%) and more Medicaid enrollees (45.2% versus 41.1%) in the group with appropriate referrals. The number of patients with private or other insurance did not significantly differ between groups (44.6% versus 44.8%). The rate of inappropriate referrals was higher for external referrals compared to internal referrals (72% vs. 28%) chi-sq, *p* = .03). There was a significantly higher percentage of patients under the age of 40 in the group that had inappropriate referrals (19.1%) than in the group with appropriate referrals (13.7) as well as in the age-group 40 to 50 (15.2% versus 13.8%).

### Regression analysis

We analyzed factors affecting appropriateness of referrals and the effect of introducing an additional rheumatologist (Table 2). We found that patient gender, severity of disease (comorbidities represented by the CCI) insurance status, and where patients live/were referred were predictive of inappropriate referrals. Females had 4.2% higher probability of inappropriate referrals than men did (*p* = 0.03). Patients with a CCI of 1 had a 15.1% lower probability (*p* < 0.01) of getting an inappropriate referral compared to those with a CCI of 0. This relationship remained for the other comparisons of non-zero CCI compared with CCI = 0. Specifically, the probability of an inappropriate referral was 16.7% lower for CCI = 2 compared to CCI-0 (*p* < .01); 13.6% lower for CCI = 3 (*p* < .01) and 18.8% for CCI = 4 and 13.9 for patients with CCI = 5 or higher (*p* < 0.01).

**Table 2:** Rheumatologist start - Base Model

	(1)		
	Inappropriate		
Start new rheumatologist	0.1475***		
	(0.0187)		
Internally referred	0.0081	Rheumatologist or DO	0.0112
	(0.0238)		(0.0332)
CCI = 1 (ref CCI = 0)	−0.1507***	Family Practice	−0.0466*
	(0.0206)	*(incl Peds, NP, PA)*	(0.0238)
CCI = 2	−0.1670***	Internal Medicine	−0.0165
	(0.0305)		(0.0235)
CCI = 3	−0.1360***	Specialized Dept.	−0.0323
	(0.0383)		(0.0278)
CCI = 4	−0.1879***	Naturopath	−0.0315
	(0.0541)		(0.0389)
CCI = 5	−0.1393***	Lamoille county	−0.1007**
	(0.0427)		(0.0444)
female	0.0419**	New Hampshire	0.2419**
	(0.0191)		(0.1073)
age	−0.0014*	New York	−0.3035**
	(0.0007)		(0.1497)
Medicaid (ref: Medicare)	0.0112		
	(0.0332)		
Private ins (ref: Mcare)	−0.0466*		
	(0.0238)		
Observations	2762		

Coefficients are marginal effects: differences in probabilities

*** *p* < 0.01, ** *p* < 0.05, * *p* < 0.1, standard errors in parentheses

Compared to Medicare enrollees, those with private or other insurance had a 4.7% lower probability of getting an inappropriate referral (*p* = 0.05). We also found that patients living in New Hampshire had a 24.2% higher probability of having an inappropriate referral (*p* = 0.02) compared to patients living in Washington county, where the hospital is based. We also found that those living in New York had a 30.4% lower probability of inappropriate referral (*p* = 0.04) compared to Washington county residents. Those living in Chittenden county, where the biggest medical center of the state is based, have a 10.1% lower probability of having an inappropriate referral (*p* = 0.02).

The addition of another rheumatologist increased the rate of inappropriate referrals. Patients who were referred when the rheumatologist started were 14.8% more likely to have an inappropriate referral compared to patients referred before the new rheumatologist started (*p* < .001). In this model, we also found that patients who were sicker and those with private insurance (compared to Medicare enrollees) were less likely to have inappropriate referrals.

**Fig. 1:**
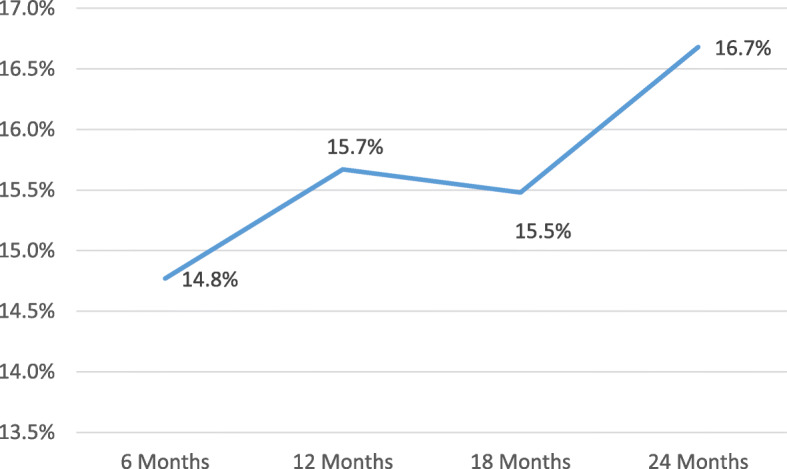
Rheumatologist -washout results (6 months, 12, 18, 24). In these 6 months, 12 months, 18 months, and 24 months-models we are controlling for the same patient and provider factors as in other models. We found that patient characteristics as well as private insurance (ref: Medicare) were significant at *p* < .05

Figure 1 shows the effect of additional supply affecting the appropriateness of referrals by time period. Inappropriate referrals increased from 14.8 to 15.0% after 6 months after the start of the rheumatologist, to 15.7% after 12 months to 15.3% after 18 months and to 16.4% after 24 months.

Table 3 shows the results of the difference-in-differences (DiD) model. The mean change in the probability of having an inappropriate referral from before to after the additional rheumatologist started was 8.6% higher among patients who were internally referred (*p* = 0.03), suggesting that the bulk of the additional inappropriate referrals were internally referred.

**Table 3:** Difference-in-Differences: start of new rheumatologist

	DiD model		
Start rheumatologist	− 0.0504	Rheumatologist or DO	− 0.0150
	(0.0372)		(0.0235)
Internally Referred	0.1176***	Family Practice	−0.0316
	(0.0230)	*(incl Peds, NP, PA)*	(0.0278)
Interaction rheumatologist*internal	0.0856**	Internal Medicine	−0.0336
	(0.0405)		(0.0391)
CCI = 1 (ref CCI = 0)	−0.1502***	Specialized Dept.	0.0091
	(0.0203)		(0.0484)
CCI = 2	−0.1658***	Naturopath	0.0359
	(0.0302)		(0.0808)
CCI = 3	−0.1362***	Lamoille county	−0.0983**
	(0.0383)		(0.0440)
CCI = 4	−0.1813***	New Hampshire	0.2433**
	(0.0540)		(0.1000)
CCI = 5	−0.1387***	New York	−0.2962**
	(0.0421)		(0.1480)
female	0.0411**		
	(0.0191)		
age	−0.0014*		
	(0.0008)		
Medicaid (ref Medicare)	0.0111		
	(0.0332)		
Private/other insurance	−0.0461*		
	(0.0240)		
Observations	2762		

Standard errors in parentheses

No significant effects for rheumatologist /DO and service line

Interaction effect goes up to 15% after 18 months and 18% after 24 months (*p* < .05)

*** *p* < 0.01, ** *p* < 0.05, * *p* < 0.1

As in the previous models, we found no significant effect for whether or not the referring provider was a physician versus APP or other type of rheumatologist.

## Discussion

In this paper, we asked what proportion of rheumatology referrals are appropriate and which factors predict the appropriateness of referrals, such as insurance type, socio-demographics, and specialty capacity (i.e., wait time). We found moderately strong agreement among the rheumatologists about which cases should and should not have been referred, and the clinician’s judgment agreed largely with the proxy EHR measure of inappropriateness.

Our findings suggest that there are high levels of inappropriate referrals and that there are systematic, modifiable factors that predict whether a referral is appropriate or not. We found that patient characteristics such as gender, age and comorbidities affect the probability of an inappropriate referral. Those who are privately insured were less likely to have an inappropriate referral than Medicare enrollees, the latter who may seek care without a referral from their primary care provider. We found no significant difference among referring physicians versus APPs and inappropriate referrals; we also did not find differences across services lines. We did find that internal referrals drive a large portion of the difference in the change in unnecessary referrals after adding another rheumatologist to the practice. This might occur because internal providers are more likely to be aware of the additional capacity than external providers are.

### Limitations

The decision to not offer a follow-up appointment may be due to other factors, such as lack of appointment slots due to limited resources. While we acknowledge that the use of a proxy of “If no continuing care was offered, then the referral was not appropriate” does not capture all inappropriate referrals, as often one appointment is all that is necessary to exclude a rheumatologic diagnosis, the study does use a new and innovative approach to evaluate referral appropriateness by comparing the results of a quantitative analysis using EHR data with the “gold standard” of chart reviews. We found there there was a strong correlation between the quantitative analysis using the proxy and the more traditional chart revi ews, suggesting that this approach should be used more often in future research.

In this study, we initially also wanted to analyze the effect of the new MD on no-show rates and cancellations, as we expected wait times to decrease and therefore no-shows and cancellations to decrease. However, we did not have enough data for this analysis and the no-show rates were generally lower than we initially expected.

## Conclusions

Reducing inappropriate referrals can happen in a multitude of ways. The challenge moving forward will be to prospectively identify the inappropriate referrals and then reduce their volume. Moving toward adopting a system where we are able to provide additional training and guidance to primary care providers may help. eConsult is a structure from which this teaching may occur [28]. Specifically, consult questions are sent to the specialist and cases are then reviewed based on the available information. Back and forth, communication can then occur between the PCP and specialist until either the issue is resolved or there is a decision to have an in-person visit. Over time, possible benefits include increased PCP knowledge, reduced wait times [29], and reduction in inappropriate in-person visits. Symptom checkers (SCs) are another potential option to accelerate diagnosis, reduce misdiagnoses, and guide patients more effectively through the health care system [30].

We also find that capacity is strongly associated with inappropriate referrals. This suggests a dilemma for the health system moving forward. As capacity was added, the vast majority of the additional referrals were appropriate, for both internal and external referrals. Yet the proportion of inappropriate referrals increased. This suggests that efforts to increase appropriate utilization through reductions in capacity may have the unintended consequence of reducing appropriate care as well. This highlights the challenges in increasing the appropriate use of high cost services as the health system transitions to value based care.

## Data Availability

The datasets used and/or analyzed during the current study available from the corresponding author on reasonable request.
